# Endoscopic detection of cancer with lensless radioluminescence imaging and machine vision

**DOI:** 10.1038/srep30737

**Published:** 2016-08-01

**Authors:** Silvan Türkcan, Dominik J. Naczynski, Rosalie Nolley, Laura S. Sasportas, Donna M. Peehl, Guillem Pratx

**Affiliations:** 1 Medical Physics, Stanford University School of Medicine, Stanford, CA 94305, USA; 2 Department of Urology, Stanford University School of Medicine, Stanford, CA 94305, USA; 3 Department of Radiology, Stanford University School of Medicine, Stanford, CA 94305, USA; 4 Department of Bioengineering, Stanford University, Stanford, CA 94305, USA

## Abstract

Complete removal of residual tumor tissue during surgical resection improves patient outcomes. However, it is often difficult for surgeons to delineate the tumor beyond its visible boundary. This has led to the development of intraoperative detectors that can image radiotracers accumulated within tumors, thus facilitating the removal of residual tumor tissue during surgical procedures. We introduce a beta imaging system that converts the beta radiation from the radiotracer into photons close to the decay origin through a CdWO_4_ scintillator and does not use any optical elements. The signal is relayed onto an EMCCD chip through a wound imaging fiber. The sensitivity of the device allows imaging of activity down to 100 nCi and the system has a resolution of at least 500 *μ*m with a field of view of 4.80 × 6.51 mm. Advances in handheld beta cameras have focused on hardware improvements, but we apply machine vision to the recorded images to extract more information. We automatically classify sample regions in human renal cancer tissue *ex-vivo* into tumor or benign tissue based on image features. Machine vision boosts the ability of our system to distinguish tumor from healthy tissue by a factor of 9 ± 3 and can be applied to other beta imaging probes.

It is well documented that better removal of residual tumor during surgery improves the outcome for patients^1^,^2^,^3^,^4^. However, it is often difficult to delineate the tumor beyond its visible boundary. A few technologies that improve the resection of tumors include microscopy, stereotactic navigation, intraoperative low-field MRI, matrix-assisted laser desorption ionization for mass spectrometry imaging (MALDI), and ultrasound imaging^5^,^6^,^7^,^8^. Progress has also been made with tracer molecules and particles that specifically target the tumor and can be interrogated optically, via ultra- sound, or by emitted radiation. Radiotracers, in particular, have experienced tremendous growth and adoption in the clinic, driven mainly by new radioimaging modalities such as positron emission tomography (PET) imaging^9^. This has lead to the development of dedicated intraoperative tools to detect radiotracers.

Intraoperative detectors and cameras that can image radiotracers specifically targeted or preferentially accumulated in tumors can facilitate the removal of residual tumor during surgical procedures. Radiotracers have already been employed for surgical guidance during the resection of breast tumors, thyroid tumors, squamous cell carcinoma, melanoma, and gliomas. Consequently, there is an interest in the development of devices that can image radiotracers in an intraoperative setting. Early imaging devices have focused on the detection of gamma radiation and have found use both in clinical trials and the clinic^10^,^11^,^12^,^13^,^14^,^15^. The disadvantage of imaging gamma radiation is that gamma rays travel many centimeters through tissue before they can be detected, leading to decreased resolution and a high level of background signal from distant tissue that may have high tracer uptake. For this reason, and further driven by the clinical development of beta emitting radioisotopes, beta particle detectors are becoming a focus of research and development for imaging in the clinical setting.

The development of beta detectors can be categorized into pure detectors, which yield a reading at a certain spot^16^,^17^,^18^,^19^,^20^,^21^, and imaging devices, which give an actual picture of the radiotracer distribution^22^,^23^,^24^,^25^,^26^,^27^,^28^. The principal need for the actual imaging of the radiotracer distribution in a sample is not for the primary detection of tumors or positive lymph nodes but for the detection of residual cancer in the surgical cavity. Scanning a beta detector across the cavity to map the radiation would require a long time and a steady hand, while imaging systems can provide a picture of the distribution with a high resolution down to 500 *μ*m^29^. The major challenges in the development of such devices are decreasing acquisition times, increasing sensitivity, and, most importantly, improving the ability to distinguish tumor from healthy tissue. Until now, such improvements were driven by a focus on developing better hardware.

We introduce an extremely simple objective-lens-free beta imaging system termed lens-less radioluminescence imaging (LRI) that uses a scintillator to convert the beta radiation emitted from radiotracers in biological tissue into photons near the decay origin. We evaluate the sensitivity and resolution of the system and compare it to other beta imaging systems. The principal advantage of our system is its imaging capability with a relatively large number of pixels and field-of-view, allowing for the application of machine vision techniques that can analyze the recorded images and extract greater amounts of clinically actionable information. We demonstrate this by automatically classifying regions of human renal cancer tissue samples as cancerous or benign based on image features *ex-vivo*. Machine vision boosts the ability of our system to distinguish tumor from healthy tissue by a factor of 9 and provides a simple means by which to improve the detection sensitivity of other beta imaging probes.

## Results

### Experimental setup of Lensless radioluminescence imaging

**Figure 1:**
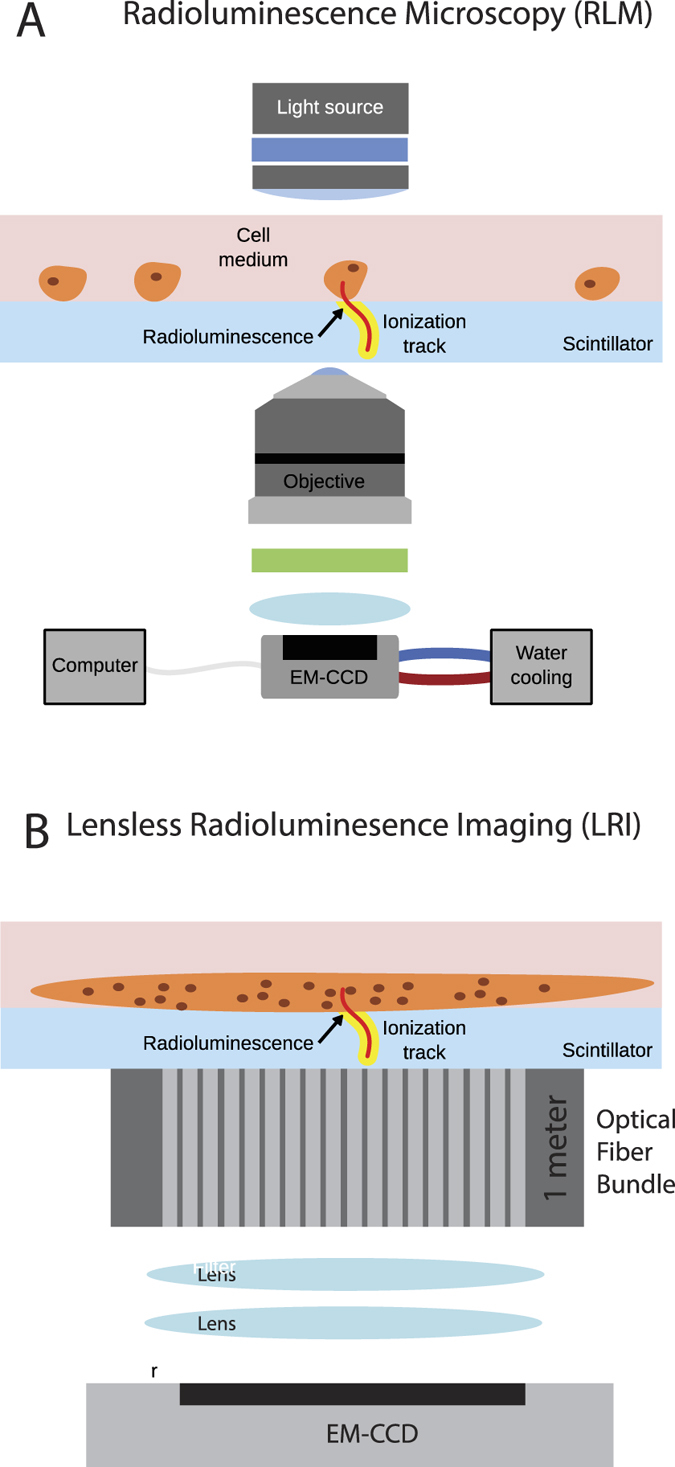
Lensless radioluminescence imaging with an endoscope. (**A**) Radioluminescence microscopy (RLM) allows for the high resolution (10 *μ*m) imaging of beta-radiation emitting radiolabeled molecules in individual cells^30^,^31^. The advantage of the technique is that decays are converted to an optical signal close to the origin of the decay, which enables decay detection with single-cell resolution. RLM utilizes a thin scintillator plate, which is in contact with the cells of interest, to convert ionizing radiation from emitted beta particles into visible-range photons detectable in a sensitive microscope. (**B**) On the other hand, lensless radioluminescence imaging (LRI) allows imaging of tissue that is in contact with the scintillator with a much greater field of view but significantly lower resolution because of the lack of an objective lens. The distribution of photons from the radiotracer is directly mapped onto the imaging chip. The compact setup allows for the addition of a fiber bundle endoscope between the sample and scintillator and the detection camera.

The details of the optical set-up are highlighted in Fig. 1. Radioluminescence microscopy (RLM) (Fig. 1a) was originally developed to achieve radiotracer detection at single-cell resolution in order to study cell-to-cell variations in tracer uptake^30^,^31^,^32^,^33^,^34^. RLM utilizes a thin scintillator plate, which is in contact with the cells of interest, to convert ionizing radiation from emitted beta particles into visible-range photons detectable by a sensitive microscope. High resolution is achieved through the magnification of the microscope objective lens. However, as a microscopy technique, RLM has a relatively small field of view (1 mm^2^). When tissue slices are imaged, even RLM cannot resolve individual cells because cells are tightly packed together. Thus for tissue imaging applications, it is more desirable to lower the resolution of the imaging technology in favor of a larger field of view. Lens-less radioluminescence imaging (LRI) is a compact optical setup that places a thin scintillator plate in contact with the tissue of interest in order to convert the locally emitted beta particles into visible-range photons (Fig. 1b). The system then directly maps these photons onto a CCD chip without an objective lens. This permits for the insertion of an imaging fiber bundle endoscope, which allows the imaging tip to be scanned over a tissue surface. The relaying lens in the optical path behind the fiber bundle does not create an image, but instead maps the light from the fibers onto the 16 *μ*m CCD chip. The relaying lens is used because we cannot bond the fibers directly onto the fragile CCD chip. This simple radioluminescence imaging setup yields a visible field of view of 4.80 × 6.51 mm with 407 × 300 pixels, which forms a true image of the radiotracer distribution in the tissue and allows for image processing. The full size of the imaging bundle is 5 × 6.7 mm, with a fiber size of 10 *μ*m. The development is motivated by a host of clinical applications using lensless microscopy in the visible spectrum^35^. The advantages for these types of imaging devices are their compact architecture and large field of view. In this work we focus on imaging the beta radiation from the ^18^F isotope in Fluorodeoxyglucose (FDG) because of its clinical importance and routine use.

### Sensitivity

**Figure 2:**
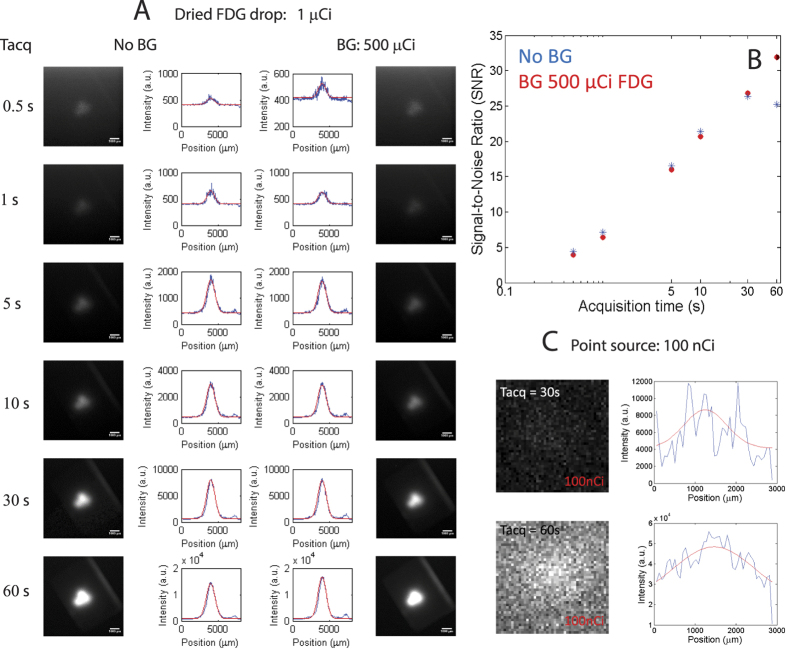
Sensitivity measurements of the LRI endoscope. (**A**) Series of signal-to-noise ratio (SNR) measurements using a dried drop of FDG (1 *μ*Ci) at different acquisition times (*t*_*acq*_) without (left) and with (right) background from gamma radiation. (**B**) Summary of SNR with respect to acquisition time. (**C**) Lower limit of detection measured with a 100 nCi pocket source.

The sensitivity of the lensless radioluminescence imaging device was evaluated in contact mode with respect to two factors, which are the signal-to-noise ratio (SNR) and detection limit. To evaluate the SNR, we placed a droplet of 1 *μ*Ci FDG on a glass slide and let the liquid evaporate to yield a 300 *μ*m spot. The spot was then imaged with the LRI system with and without a background FDG sample of 500 *μ*Ci directly behind the glass slide to provide a background of gamma radiation. This image series is shown in Fig. 2a for a range of acquisition times (*t*_*acq*_). The system was able to detect the dried 1 *μ*Ci spot even at an *t*_*acq*_ of 0.5 s with a SNR of 4. The SNR for all images is shown in Fig. 2b. To evaluate a lower limit of detection, we used a 51nCi ^204^Ti pocket point source 2c. The LRI system can still detect the point source using a *t*_*acq*_ of 60 s. The lower detection efficiency (≈17%) of the point source with respect to the dried FDG is probably due to the fact that the emitting isotope cannot be placed in direct contact with the scintillator because of the source geometry.

### Resolution

**Figure 3:**
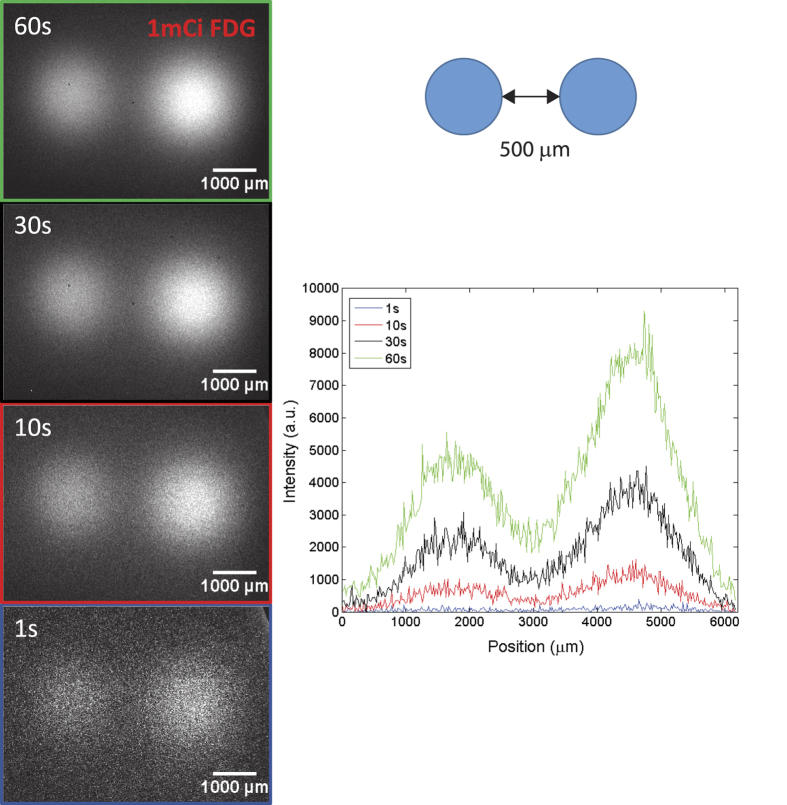
Resolution of the LRI endoscope. Series of images from two dried drops of FDG (1 *μ*Ci), which are separated by 500 *μ*m at different acquisition times (*t*_*acq*_). The line scan along the row of the maximum pixel is shown for the different *t*_*acq*_ using the color code in the image frames. Both spots can be resolved down to an *t*_*acq*_ of 1 s.

The resolution of the LRI system was tested by placing two drops of dried FDG (1 *μ*Ci) at a distance of 500 *μ*m. The contact mode images in Fig. 3 show that both spots can be clearly distinguished giving an upper estimate for the resolution. The full width half maximum (FWHM) of a Gaussian fit to the image of the pocket source gives a spot of 300 *μ*m for the 51nCi ^204^Ti point source.

### Automated classification of tissue

**Figure 4:**
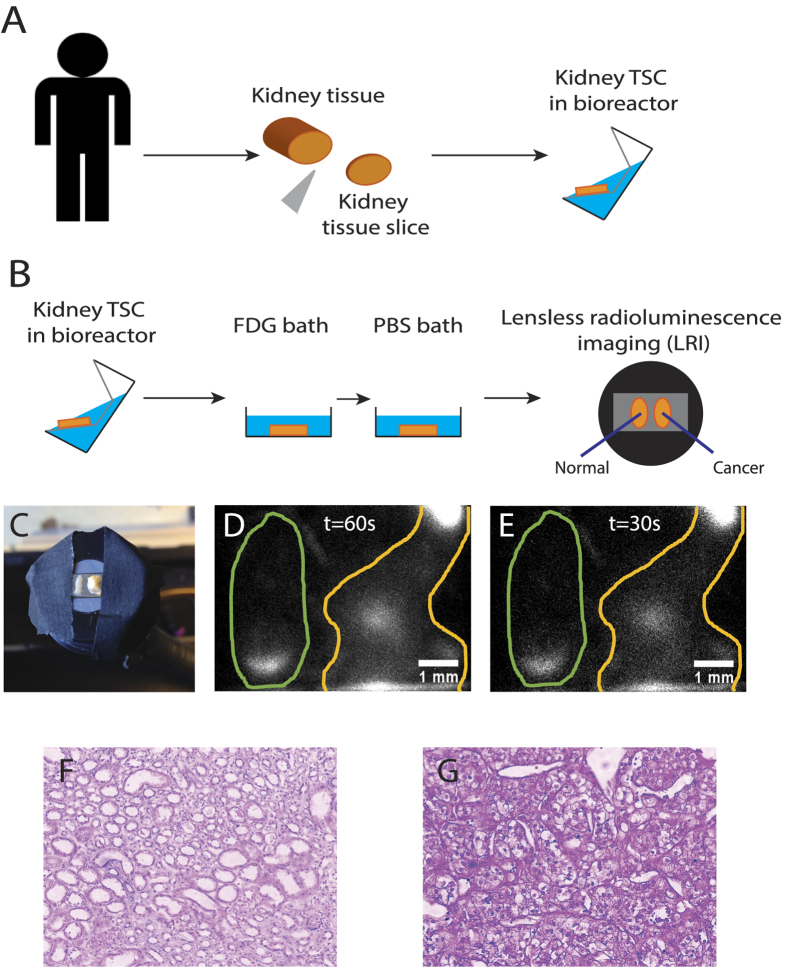
LRI imaging of human tissue slice cultures (TSCs) of renal cell carcinoma and benign kidney tissue. (**A**) Surgical removal of cancer tissue and preparation of kidney TSCs. (**B**) Experimental method for imaging FDG uptake in human renal TSCs. (**C**) LRI endoscope with a TSCs of benign kidney (left) and RCC (right). (**D,E**) Images of the distribution of FDG in the TSCs with an acquisition time of 60 *s* and 30 *s*, respectively. (**F**) H&E stain of normal kidney sample. (**G**) H&E stain of tumor samples shows clear cell renal carcinoma.

The strength of the LRI endoscope system is the imaging capability with respect to the large amount of pixel per image and the resolution of 500 *μ*m. To demonstrate this, we imaged human renal tissue slice cultures (TSCs) from patients undergoing resection of renal cell carcinoma (RCC). Cores of normal kidney and RCC were obtained and maintained as thin, precion-cut TSCs overnight. Before imaging, the tissue was starved of glucose and incubated with FDG *ex-vivo* as shown in Fig. 4a,b.

**Figure 5:**
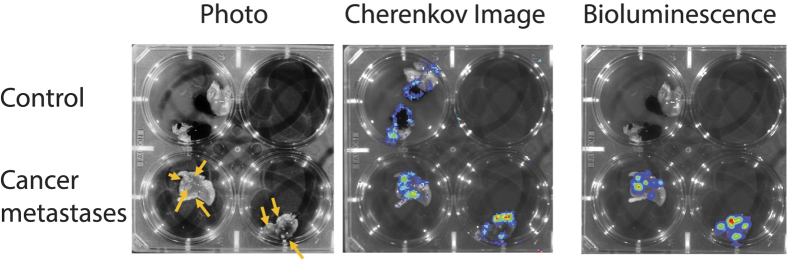
FDG uptake correlates with cancer. Bioluminescent 4T1 cancer cells were grown in the mammary fat pad of mice and the cancer was allowed to grow until it metastasized into the lung. Lung metastases are highlighted by the yellow arrows in excised lungs from two animals. The control animal, shown in the top row, did not have a primary tumor and consequently no metastases. Lungs were subsequently incubated in FDG under the same conditions as the human TSCs. FDG is directly imaged using Cherenkov imaging in an IVIS system. High intensity regions in the Cherenkov image are due to a high local uptake in FDG. The control lung without cancer shows low levels of Cherenkov light at the edges, which may be an artifact of FDG accumulation due to drying. The bioluminescence image of the 4T1 cells (cancer cells) in the lung shows the location of the metastatic 4T1 cancer cells. Regions of cancer metastases correlate well with the FDG uptake that is determined by Cherenkov imaging. This shows that *ex-vivo* incubation with a radiotracer is a valid procedure similar to *in-vivo* radiotracer uptake.

To verify if FDG accumulation occurs in *ex vivo* cancer tissue during incubation with radiotracer, we performed murine engraftment studies using bioluminescent 4T1 breast cancer cells. Bioluminescent 4T1 cells were injected into the mammary fat pad of two mice and tumors were allowed to develop until metastasis were detected in the lung. Following excision, the lungs were incubated *ex-vivo* in FDG and rinsed in PBS. Lung metastases are highlighted by the yellow arrows in excised lungs from two animals (Fig. 5). The lung of a control animal without tumor is shown in the top row for comparison. FDG was directly detected by Cherenkov imaging in an IVIS system. High intensity regions in the Cherenkov image are due to a high local uptake in FDG. The control lung without 4T1 metastases shows low levels of Cherenkov light at its edges, likely an artifact of FDG accumulation due to drying. Notably, there was complete absence of signal within the tissue edges. To confirm the presence of 4T1 metastases, bioluminescence imaging of the lung sample was performed. Regions of cancer metastases identified by bioluminescence correlated well with FDG uptake determined by Cherenkov imaging, validating the *ex-vivo* radiotracer incubation procedure.

Next, we assessed the performance of LRI to image tumor burden in clinical tissue samples. Samples of renal tissue from patients undergoing nephrectomy were prepared as thin tissue slice cultures (TSCs) and exposed to FDG (Fig. 4a). Following incubation, samples of both healthy and tumor burdened renal tissue were positioned side by side on the endoscope and prepared for LRI (Fig. 4c,d). LRI revealed a clear signal in the renal cancer sample resolved with acquisition times of 60 and 30 s (Fig. 4d,e). A standard intensity value measurement (I_*max*_) shows values of 940 for the normal tissue and 1205 for the cancer tissue. Similarly, the mean uptake value (I_*mean*_) is 300 ± 20 for the normal tissue and 490 ± 20 for the cancer tissue. Although the cancer displays quantitatively higher I_*max*_ and I_*mean*_ values, the difference remains small and may not be distinguishable in a patient.

**Figure 6:**
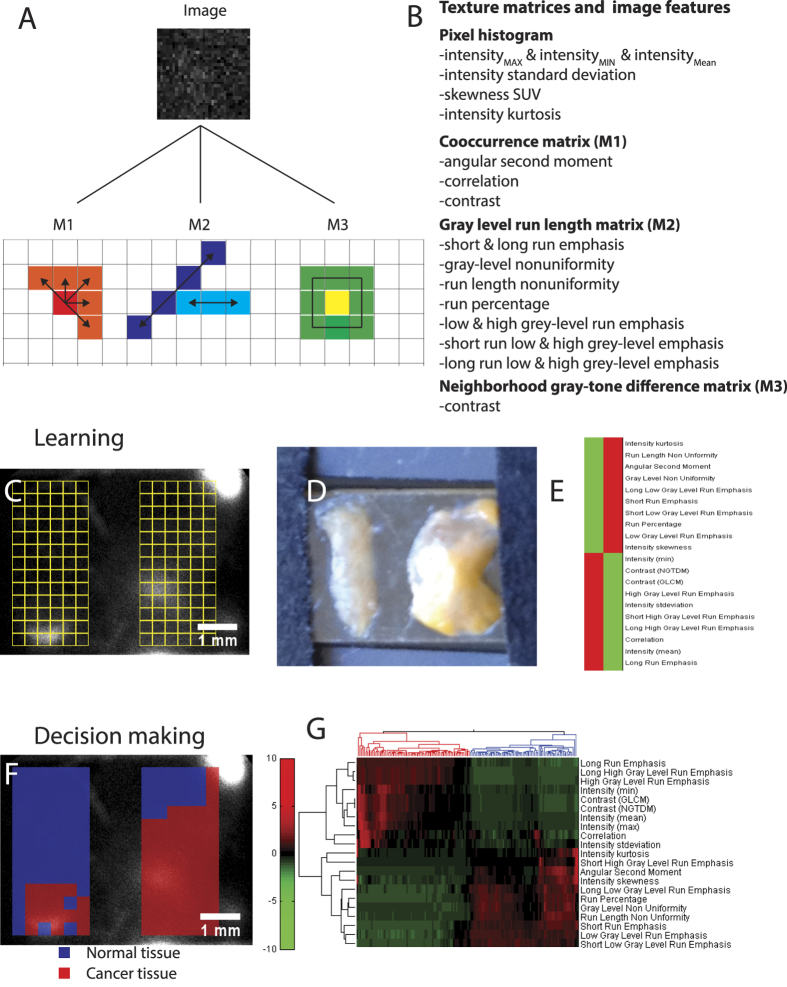
Machine vision for the classification of tumor and benign tissue. (**A**) 22 image features are used for classification. All features are calculated from the global image (pixel histogram) or one of three types of image matrices (M1–3) that capture local and regional features. (**B**) Image matrices and 22 associated features used for classification. (**C,D**) LRI endoscope with a tissue slice culture of normal kidney (left) and renal cancer (right). Selected ROIs on both samples are shown in yellow. Small ROIs are 20 by 20 pixels. (**E**) Computed and clustered image features for all ROIs show the image features that are larger in the tumor in red (Mean C) and larger in normal tissue (Mean N). (**F**) Automated classification of small ROIs using clustering of image features knowing which parameter are larger in the tumor or normal tissue from (**C**). (**G**) Clustering result of the small ROIs with normal (blue) or tumor (red). Seventeen out of 78 ROIs are misclassified in the normal and in the cancer tissue.

This ambiguity in decision making between cancerous and normal tissue motivated the application of machine vision to images generated with LRI. The use of machine vision in LRI is possible because of the large number of pixels and resolution limits afforded to this approach. Textural analysis of image features was achieved by following the the work of Tixier *et al*.^36^ in 2 dimensions through the resampling of pixels in a region of interest (ROI), followed by computing three different texture matrices (M1–3) or by analyzing all pixels in the ROI. The three texture matrices capture local and regional textures by describing how a single pixel or a group of pixels differ with respect to their surroundings. The pixel statistics of the entire ROI captures global texture. The cooccurrence matrix (M1) is a local measurement that characterizes texture by means of second order statistics and, captures how the pixels immediately surrounding a pixel differ (Fig. 6a)^37^,^38^. We calculate three features from this matrix, which are listed in Fig. 6b. The gray level run length matrix (M2) describes the occurrence of runs for each gray level and length in different directions^39^,^40^,^41^. The 11 features calculated from this matrix are listed in Fig. 6b. Finally, the neighborhood gray-tone difference matrix (M3) is a local measure of texture that captures how groups of pixels of a certain gray scale immediately surrounding a pixel differ, providing an additional feature^36^,^41^. This matrix gives us an additional feature. Together, these 21 image features allow us to classify regions of interest.

First, we start by defining 6 × 13 ROIs that cover most of the tissues of interest, one in the cancer sample and one in the normal tissue sample of the image (Fig. 6c). Automated clustering based on these features measured over the entire group of ROIs highlights how cancer tissue differs from normal tissue in terms of these features. As previously observed with the normal intensity analysis, the cancer tissue shows a higher intensity (*I*) (I_*mean*_ & I_*min*_ & I_*max*_). However, machine vision now offers an additional 18 features that highlight the difference between cancer and normal tissue (Fig. 6e). Notably, cancer tissue shows greater contrast and gray level run emphasis, while normal tissue is more uniform.

Using the image features, we can now use machine vision to automatically classify the smaller ROIs that are outlined in Fig. 6c for the sample arrangement shown in 6d. The automated classification is shown in Fig. 6e, where a red outline indicates group 1 and a blue outline indicates group 2. We know that the cancerous tissue was placed on the right, so we can rename the red group 1 as cancer and the blue group 2 as normal tissue. This automated classification is based on the clustering of image features that is shown in Fig. 6g. The Z-score values used for clustering of the individual ROIs in Fig. 6g are shown in Supporting Fig. S1. This approach misclassified 17 out of 78 normal regions as cancer (false positives) and 17 out of 78 cancer regions as normal (false negatives). This translates into a false positive rate of 22% and a false negative rate of 22% for the shown sample. In all five imaged samples the false positive rate was 21 ± 3% and the false negative rate was 23 ± 2% (Table S2). The misclassified false positive ROIs of are likely due to the relatively high local signal variation, which arises due to the accumulated FDG at the edge of the tissue. The clustergram also indicates that prior knowledge of where cancer and normal tissue is are not required. Clustering the image features will automatically yield groups and show which ROIs are similar.

## Discussion

The development of intraoperative beta probes is driven by the premise of achieving better patient outcomes following surgical resection of tumors by more accurately distinguishing residual tumor presence. This technology is especially important for the treatment of cancers such as glioma, where precisely determining tumor margins has been shown to improve prognosis following surgery and handheld beta probes in conjunction with new probes allow definition of the lesion boundary beyond the visible tumor boundary^19^,^42^,^43^,^44^. Beta probes can be divided in detectors and probes that have imaging capabilities. The advantage of the imaging capabilities of the probe is a better resolution and it is crucial for the detection of small tumors where scanning probes becomes time consuming.

We have developed a relatively simple beta particle imaging technique called LRI, based on a CdWO_4_ scintillator crystal, which is directly coupled to a wound imaging fiber bundle with 10 *μ*m fibers in a 5 by 6.7 mm bundle. The handheld fiber bundle allows the system to be flexible and mobile, enabling intraoperative imaging of tissue by simply scanning its surface and mapping the signal onto a EMCCD chip. LRI can detect a source with an activity down to 100 nCi or 3700 Bq with an acquisition time of 60 s. Higher activity allows the imaging with acquisition times down to 5 s. Other beta cameras with imaging capabilities allow imaging of 100 nCi^24^, 2 nCi^29^, and 5000 nCi^28^ with acquisition times ranging from 2 to 30 s. Our sensitivity is thus comparable to other imaging beta cameras, whose slightly better performance is achieved by detecting beta and gamma emissions from the sample. Cameras such as these require subtracting the background gamma emission in order to improve image quality. The background gamma emission did not pose a problem to our relatively simple imaging system, as shown in Fig. 2, where the SNR of our system was not impacted by the background gamma radiation from an activity of 500 nCi. Overall, the LRI system’s SNR is comparable with other handheld beta cameras that use more sophisticated engineering to improve light guiding to improve detection efficiency or background subtraction^28^,^29^.

The resolution of the LRI system presented here is at least 500 *μ*m with a field of view of 4.80 × 6.51 mm, which we determined by clearly resolving two dried FDG spots of 1 *μ*Ci. A handheld device with a CsI:Ti scintillator coupled to a compact silicon photomultiplier achieves 1.5 mm^24^ with a field of view of 10 × 10 mm^2^. Previous iterations of scintillators coupled to a photomultiplier have shown a resolution of 500–600 *μ*m with a field of 1.2 cm^2^ 22,25,26,27,28,29.

The purpose of handheld beta cameras is to assist surgeons in identifying residual tumor during surgery and guide resection. A measure of the ability to distinguish tumor from normal tissue is to measure the tumor to normal ratio (TNR) in terms of measured signal strength. Non-imaging beta cameras that only detect beta radiation and have poor resolution, can achieve TNRs of 3.5^19^, 3–10^20^, 1.24–3.4^21^, 2.7–6.3^45^, and 6.6^17^. Tipnis *et al*. demonstrated a TNR of 10 using an imaging system probe that subtracts gamma radiation from the acquired signal. In their work, tumor phantom had an activity 12 times that of the background phantom. However, TNRs in cancer patients vary between 1.3 and 16. Our TNR of the shown sample of 2.1 was obtained by comparing the mean FDG uptake in a human RCC cancer TSC and comparing it to a normal renal TSC, which is above a ratio of 1.5. The average TNR of all samples is 3 ± 1. A TNR of 1.5 is considered to be required for tumor detection^17^. However, we believe that this ratio is not stringent enough to robustly aid the surgeon in determining residual cancer. Therefore, we have added machine vision to better process the images taken by handheld beta cameras.

Recent advances in handheld beta cameras have focused on hardware improvements and have produced devices with imaging capabilities that greatly increase spatial resolution. However, increasing resolution through image analysis has not yet been explored. Processing captured images using image analysis techniques can potentially add significantly more value to captured images. Machine vision is an easily implemented process that can aid in the classification of clinical images, as has been shown in predicting the response to concomitant radiochemotherapy in esophageal cancer using PET imaging^36^. We used the global information from all pixels in the image and three texture matrices: cooccurrence matrix, gray level run length matrix, and neighborhood gray-tone difference matrix. The resulting 21 image features can distinguish well between cancer and normal tissue for our clinical renal samples (6d). In general, cancer tissue displays more contrast, correlation, and high gray level run emphasis along with an overall higher FDG signal. Automated clustering of smaller ROIs shows how this endoscope could be operated in a decision making process (6f,g). ROIs in the cancer and normal tissue group strongly. Seventeen out of 78 normal ROI are misclassified into cancer (false positives), which is most likely caused by excess FDG runoff at the edge of the tissue. Using the 21 features greatly increases the TNR because we now added much higher dimensionality to the data compared to only using mean counts. The TNR could now be calculated by combining the TNR of all of the 16 independent image features out of the total 21 image features. This improves the TNR of 2.1 of the initial system to a TNR of 18, simply by extracting more information from the acquired data using machine vision (Table S3). The TNR from the independent image features of all samples amounts to 26 ± 19 and translates into a 9 ± 3 fold improvement in TNR by using image features (Table S4). This approach for improving the TNR is easy to implement and can be applied to other beta imaging probes. Adopting a machine vision procedure can further improve clinically important improvements by switching from the 1.5–1 ratiometric threshold criteria for distinguishing disease from normal tissue to the three-sigma statistical threshold criteria for this new class of imaging based beta probes^46^. The current classification of cancer and normal tissue and the TNR can be improved by realizing that the applied clustering based on Z-values assumes a Gaussian distribution, which is a good first estimate, but certainly an oversimplification. Certain image features have non-Gaussian distributions and may not take on negative values. The use of a folded Gaussian or other models for describing the distribution of image features in the ROIs will improve the clustering efficiency and can reduce the rate of false positives and false negatives and are an avenue for improving the technology.

## Methods

### Lensless radioluminesence imaging (LRI)

LRI utilizes a 10 × 10 × 0.5 mm CdWO_4_ scintillator crystal (MTI Corporation), which is in contact with the tissue of interest, to convert ionizing radiation from emitted beta particles of tracers in the tissue into visible-range photons detectable in a sensitive microscope. The collected photons pass through a wound fiber bundle (IG-567, Schott AG) and are mapped onto a camera chip via a relaying lens (Schott AG). The wound fiber bundle allows motion of the imaging tip while the camera stays in place. The fiber bundle has a 40% transmission between 500 and 1200 nm, a numerical aperture of 0.63, and length 1.720 m. The 5 by 6.7 mm fiber bundle has 10 *μ*m elements in a 6 × 6 array. The camera is a EMCCD (ProEM, Princeton Instruments, Trenton, NJ) with a 512 × 512 imaging chip with a pixel size of 16 *μ*m. The imaging setup yields a visible field of view of 4.80 × 6.51 mm with 407 × 300 pixels. Images were analyzed using Matlab R2012b (Mathworks).

### Renal cancer tissue preparation

Fresh renal cell carcinoma (RCC) and normal kidney tissues were obtained from patients undergoing nephrectomy at Stanford with informed consent. All human tissue studies were conducted in accordance with and were approved by the Stanford University institutional review board of the Research Compliance Office (RCO). We only used approved protocols in this work. Cores from nephrectomy specimens were prepared with an automated coring device (Alabama Research and Development, Mundford, AL) under aseptic conditions before precision-cutting of tissue into 300 *μ*m thick tissue slices was performed with a Krumdieck tissue slicer (Alabama Research and Development) as previously described^47^,^48^,^49^. Frozen sections were utilized to histologically confirm the diagnosis of RCC and benign histology. The tissue slices were placed on grids in 6-well dishes containing Complete PFMR-4A medium and incubated overnight on a rotary apparatus in a 37 degree 5% CO_2_ incubator^48^. Before imaging, the tissue was starved of glucose for 45 minutes and incubated with FDG (100 *μ*Ci/mL) for 15 minutes *ex-vivo*. TSCs were then rinsed three times in PBS.

### Animal Model

All animal studies were conducted in accordance with and were approved by the Stanford University Institutional Animal Care and Use Committee (IACUC) and used approved protocols. Female Nu/nu mice were purchased from Charles River Laboratories (Wilmington, Massachusetts). 1*x*10^6^ bioluminescent 4T1 breast cancer cells^50^ were freshly harvested and resuspended in a 100 *μ*L solution containing 50% culture medium and 50% Matrigel (BD Biosciences). The suspension was then orthotopically implanted by injection into the 4^*th*^ left mammary fat pad and tumors were grown over 3 weeks.

### Cherenkov and bioluminescence imaging of lungs

Cherenkov and bioluminescence imaging were performed with an optical imaging system (Xenogen IVIS 200) and an acquisition time of 60 s. Following Cherenkov imaging, we added luciferin to trigger bioluminescence, which was imaged with an acquisition time of 5 s.

### Radiotracer (FDG) production

The glucose analogue FDG was prepared from mannose triflate precursor via nucleophilic 18F-fluorination and hydrolysis. The ^18^F was produced in a GE PETtrace cyclotron. The production was performed on a cassette based automated synthetic module (FASTlab, GE Healthcare). Quality control criteria were set and the tests were performed according to USP823. Because of its short lifetime, FDG was used within 8 h after it was produced and dosed at the levels described below for experiments. The radioactivity was measured with a dose calibrator.

### SNR calculation

The signal to noise ratio (SNR) was calculated by dividing the amplitude *A* of the signal by the square root of the square of the standard deviation *σ*_*B*_*G* of the background intensities plus the amplitude *A* (shot noise) using:


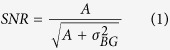


The amplitude was determined by subtracting the image background and applying a Gaussian fit to the signal. The standard deviation of the background intensities was measured on a ROI that did not contain signal.

### Calculation of image features

We used the global information from all pixels in the ROI and three texture matrices: cooccurrence matrix, gray level run length matrix, and neighborhood gray-tone difference matrix. All the pixels in the selected ROI were analyzed to give values for the global maximum pixel (Intensity_*max*_), minimum pixel (Intensity_*min*_), and mean pixel value and standard deviation (Intensity_*mean*_ & Intensity stdeviation). We also calculated skewness and kurtosis from the global pixel histogram. The pixels in the ROI are then resampled giving each pixel *P* a value using:


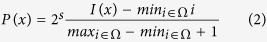


where 2^*s*^ represents the number of discrete values (32 in our case), *I* is the original intensity of that pixel, and Ω is the set of pixels in the ROI.

Now we computed the cooccurrence matrix (M1) using:


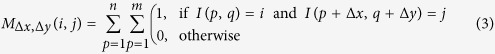


where *M*_Δ*x*,Δ*y*_(*i, j*) is the *i*th and *j*th entry in the cooccurrence matrix parametrized by an offset (Δ*x*, Δ*y*) of an *n* by *m* image. We used the graycomatrix function of Matlab and chose to calculate the cooccurrence matrix along 4 directions of distance 1 and average the 4 matrices to get a single cooccurence matrix. We calculate three image features from this matrix:

Angular Second Moment:


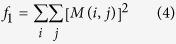


Correlation:


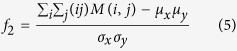


Contrast:


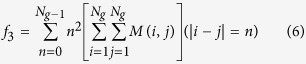


The next texture matrix we compute is the gray level run length matrix (M2)^39^,^40^,^51^. We used the routine (http://www.mathworks.com/matlabcentral/fileexchange/17482-gray-level-run-length-matrix-toolbox/content/GLRL/grayrlmatrix.mgrayrlmatrix.m) provided by Xunkai Wei for Matlab^52^. The matrix element (i, j) specifies the number of times that the picture contains a run of length j, in the given direction, consisting of points having gray level i. We calculate the matrix for runs in all for directions. The 11 extracted image features are well described in ref. 53.

The last matrix we use is the neighborhood gray-tone difference matrix (M3)^38^,^54^, which is actually a column vector and calculated by evaluating 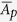 for a pixel *p* with coordinates (k, l) for a square neighborhood of size *d* using


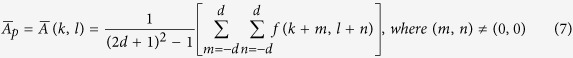


The matrix is then calculated by


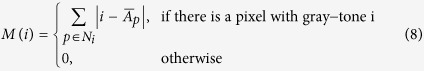


where *N*_*i*_ is the pixel with gray tone i. The calculated two features are:

Contrast:


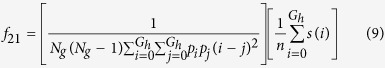


where *G*_*h*_ is the largest gray-tone in the ROI and *p*_*i*_ = |*N*_*i*_|/*n* and *n* = (*width*_*ROI*_ − 2*d*) (*height*_*ROI*_ − 2*d*). *N*_*g*_ is the number of different gray-tones present in the image.

### Calculation of image features

After calculating all 21 image features for at least two ROIs, we use automated clustering of the ROIs using the Z-score of the features, which is calculated from the average and standard deviation of the values of a specific feature across all ROIs in the analysis. The Z-scores are clustered into two clusters along rows (features), then columns (ROIs) based on their Euclidian distance. We used the clustergam routine in Matlab to generate the clustergams.

## Additional Information

**How to cite this article**: Türkcan, S. *et al*. Endoscopic detection of cancer with lensless radioluminescence imaging and machine vision. *Sci. Rep.* **6**, 30737; doi: 10.1038/srep30737 (2016).

## Supplementary Material

Supplementary Information
