# Supplementary material for: Endoscopic detection of cancer with lensless radioluminescence imaging and machine vision
Source: Sci Rep. 2016 Aug 1;6:30737. doi: 10.1038/srep30737 (PMC4967900; doi:10.1038/srep30737)
Supplement: Supplementary Information [file srep30737-s1.pdf]

Supplementary Information for

Endoscopic detection of cancer with lensless radioluminescence imaging and machine vision

Silvan Türkcan[1], Dominik J Naczynski[1], Rosalie Nolley[2], Laura Sasportas[3,4], Donna M Peehl[2], Guillem Pratx[1,*]

[1]Medical Physics, Stanford University School of Medicine, Stanford, CA 94305, USA

[2]Department of Urology, Stanford University School of Medicine, Stanford, CA 94305, USA

[3]Department of Radiology, Stanford University School of Medicine, Stanford, CA 94305, USA

[4]Department of Bioengineering, Stanford University, Stanford, CA 94305, USA

*Correspondence: pratx@stanford.edu

Figure S1

Table S2

Table S3

Table S4

A

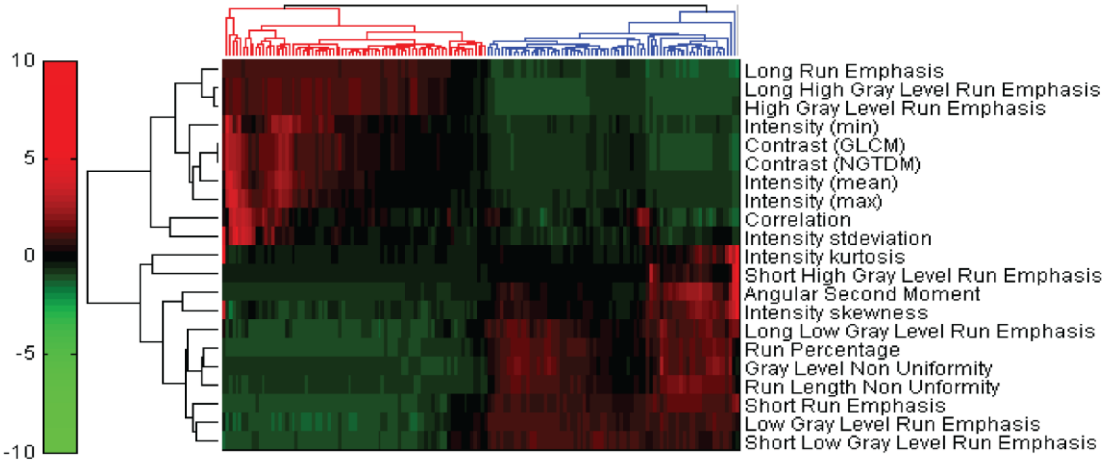

B

Z-Score ([Value-Mean]/StdDev)

	Cancer	Cancer	Cancer	Cancer	Cancer	Cancer	Normal	Normal	Normal	Normal	Normal	Normal
'Long Run Emphasis'	1.22	1.22	1.08	1.09	0.71	-0.28	-0.62	-0.85	-0.97	-0.45	-1.14	-1.17
'Long High Gray Level Run Emphasis'	1.32	1.35	1.11	1.11	0.70	-0.56	-0.86	-0.96	-0.98	-0.67	-1.00	-1.01
'High Gray Level Run Emphasis'	1.32	1.35	1.11	1.11	0.70	-0.58	-0.87	-0.98	-0.98	-0.68	-0.99	-1.01
'Intensity (min)'	4.38	2.23	1.11	-0.05	0.32	-0.39	-0.80	-0.70	-0.85	-0.47	-0.69	-0.81
'Contrast (GLCM)'	3.22	1.85	1.03	0.58	-0.14	-0.40	-0.39	-0.83	-0.81	-0.35	-1.06	-0.91
'Contrast (NGTDM)'	3.22	1.85	1.03	0.58	-0.14	-0.40	-0.39	-0.83	-0.81	-0.35	-1.06	-0.91
'Intensity (mean)'	3.68	2.35	0.81	0.17	-0.05	-0.47	-0.52	-0.75	-0.73	-0.46	-0.86	-0.82
'Intensity (max)'	3.08	1.74	0.37	-0.05	-0.03	-0.49	-0.23	-0.72	-0.65	-0.67	-0.84	-0.72
'Correlation'	-0.28	1.84	-0.56	0.02	-0.38	-0.78	0.86	-0.70	-0.20	0.57	-0.99	-0.45
'Intensity stdeviation'	0.53	0.38	-0.14	-0.60	-0.30	-0.34	0.20	-0.64	-0.47	-0.46	-0.67	-0.17
'Intensity kurtosis'	-0.51	-0.71	-0.54	-0.53	-0.50	-0.66	-0.09	0.13	-0.18	-0.65	0.67	1.36
'Short High Gray Level Run Emphasis'	-0.52	-0.52	-0.54	-0.54	-0.52	-0.45	-0.09	-0.16	0.21	-0.35	1.26	0.51
'Angular Second Moment'	-0.81	-0.82	-0.72	-0.72	-0.59	-0.48	-0.39	0.35	0.38	-0.52	1.59	1.14
'Intensity skewness'	-0.98	-0.91	-1.17	-0.65	-1.03	-0.78	0.08	0.54	0.37	-0.34	1.20	1.48
'Long Low Gray Level Run Emphasis'	-1.23	-1.01	-0.99	-1.17	-1.10	-0.24	1.07	0.88	0.62	0.02	1.26	1.22
'Run Percentage'	-1.02	-1.02	-0.99	-0.99	-0.92	-0.28	0.49	1.08	1.01	-0.12	1.15	1.51
'Gray Level Non Uniformity'	-0.85	-0.85	-0.90	-0.90	-0.97	-0.57	0.54	1.14	0.79	-0.37	1.01	1.68
'Run Length Non Unformity'	-0.92	-0.92	-0.91	-0.91	-0.93	-0.50	0.02	1.03	0.90	-0.29	1.59	1.47
'Short Run Emphasis'	-1.11	-1.11	-1.12	-1.12	-0.70	0.21	0.48	0.89	0.93	0.33	1.30	1.14
'Low Gray Level Run Emphasis'	-1.33	-1.22	-1.10	-1.18	-0.82	0.39	1.07	1.08	0.82	0.55	0.92	1.12
'Short Low Gray Level Run Emphasis'	-1.18	-1.18	-1.20	-1.20	-0.51	0.82	0.81	1.27	0.91	0.80	0.83	0.91

Figure S1: Clustering of ROIs based on the Z-Scores of the image features. (a) Clustering result of the small ROIs in Fig. 6c based on the feature. Red shows higher than mean values calculated from the Z-score, while green represents below average values. (b) The Z-scores of some of the matrix elements in the clustergram in a.

	Cancer ROIs	False negatives	Normal ROIs	False positives
Sample 1	78	17 (22%)	78	61 (22%)
Sample 2	52	12(23%)	52	11 (21%)
Sample 3	62	16 (26%)	62	16 (25%)
Sample 4	78	16 (21%)	78	14 (18%)
Sample 5	44	10 (23%)	44	9 (21%)
Mean		**23±2%**		**21±3%**

Table S4: Performance in tumor samples. We measure the performance of the imaging system in 5 tumor samples.

	Mean C	Mean N	TNR
min	31.84	12.08	2.64
max	43.32	25.26	1.71
mean	37.03	17.25	2.15
stdeviation	2.43	2.43	1.00
skewness	0.59	1.21	0.49
kurtosis	3.70	4.97	0.74
Short Run Emphasis	0.14	0.35	0.40
Long Run Emphasis	860.44	639.92	1.34
Gray Level Non Uniformity	18.24	44.59	0.41
Run Length Non Unformity	39.30	65.06	0.60
Run Percentage	0.10	0.22	0.46
Low Gray Level Run Emphasis	0.24	0.48	0.50
High Gray Level Run Emphasis	163.25	52.60	3.10
Short Low Gray Level Run Emphasis	0.11	0.23	0.50
Short High Gray Level Run Emphasis	0.49	2.14	0.23
Long Low Gray Level Run Emphasis	109.43	232.66	0.47
Long High Gray Level Run Emphasis	166807.29	51690.14	3.23
Angular 2nd Moment	0.06	0.22	0.29
Contrast (GLCM)	4.22	2.25	1.88
Correlation	0.26	0.27	0.96
Contrast (NGTDM)	4.22	2.25	1.88

SUM:	17.51

Table S3: Tumor to normal ratio from machine vision. We show the tumor to normal (TNR) for each of the image features. Summing up the features gives improves the TNR from the initially 2.1 to 17.51. This is an 8 fold increase.

Sample	1	2	3	4	5
TNR simple	2.1	3.5	2.2	4.6	1.9
TNR features	17.51	20.45	17.73	59.20	14.12
Fold improvement	8.3	5.8	8.2	12.7	7.6

Mean	STD
2.9	1.2
26	19
9	3

Table S4: Fold improvement in TNR for all samples. The average fold improvement in TNR from the image features is 9±3 for all samples.
